# Vitamin D level and it is association with the severity of pulmonary tuberculosis in patients attended to Kosti Teaching Hospital, Sudan

**DOI:** 10.3934/microbiol.2020004

**Published:** 2020-03-13

**Authors:** Samah Sidahmed M.S Elsafi, Bakri Mohammed Nour, Adam Dawoud Abakar, Izzedeen Haroun Omer, Babiker Saad Almugadam

**Affiliations:** 1 Department of Medical Microbiology, Kosti Teaching Hospital, Kosti, Sudan; 2 Department of Medical Parasitology, Faculty of Medical Laboratory Sciences, University of Gezira, Wad Medani, Sudan; 3 Department of Parasitology, Blue Nile National Institute for Communicable Diseases, University of Gezira, Sudan; 4 Department of Medicine, Faculty of Medicine, University of El Imam El Mahdi, Kosti, Sudan; 5 Department of Medical Microbiology, Faculty of Medical Laboratory Sciences, University of El Imam El Mahdi, Kosti, Sudan

**Keywords:** *Mycobacterium tuberculosis*, HIV, Rifampicin, tuberculosis, vitamin D deficiency

## Abstract

Globally, tuberculosis is one of the major causes of morbidity and mortality in many countries. Previous studies suggest that the incidence and severity of tuberculosis are associated with low levels of vitamin D (Vit D). Therefore, this study aimed to determine the occurrence and associated factors of vitamin D3 deficiency in pulmonary tuberculosis patients at White Nile State, Sudan. 101 individuals of diagnosed pulmonary tuberculosis patients (71 males and 30 females) and 100 non-TB controls (58 males and 42 females) were included in this study. Sputum samples were obtained from TB patients and subjected to examination for acid-fast bacilli (AFB) using Ziehl-Neelsen (ZN) stain and Gene Xpert analysis. Blood samples were collected from both groups and Serum 25(OH)-vitamin D3 was determined by an Enzyme-Linked Immunosorbent Assay. HIV infection in Tuberculosis (TB) group was also investigated using the immunochromatographic test. In our study, the majority of TB patients were suffered from TB relapse (36.6%); non-HIV infected individuals (99.1%) or showed a positive result for AFB (61.4%) in Gene Xpert analysis. Moreover, there is a significant difference in microscopy findings and bacillary levels of AFB, and Rifampicin (RIF) susceptibility pattern of *M. tuberculosis* strain among sputum samples of TB patients, P-values less 0.0001. Furthermore, we found that TB patients were suffered from vitamin D deficiency. In particular, the mean of vitamin D level was significantly much lower in TB patients (26.7 ± 1.6) compared to non-TB controls (117.3 ± 3.2), P-value equal 0.0001. Likewise, it's much lower in females, individuals of 21–40 years old, and patients with high bacillary levels or those infected by Rifampicin resistance strain. Accordingly, our study was highlighted the TB and Vit D deficiency relationship and showed the need for further studies to a better understanding of the impact of TB on Vit D level and investigate whether vitamin D supplementation can have a role in the prevention and treatment of tuberculosis.

## 1. Introduction

Tuberculosis (TB) is a major public health problem and cause of mortality globally [1]. TB caused mainly by *M. tuberculosis* that most often affects lungs leading to pulmonary tuberculosis. The host susceptibility to tuberculosis infection depends on a complex interaction between host, bacteria and several factors such as poverty, malnutrition, overcrowding, and exposure to other pathogens. Previously, it has been estimated that about one-third of the world's population is infected with latent *M. tuberculosis* [2], and only 10% of which will develop the active disease [3]. Approximately more than 95% of the estimated cases and deaths due to tuberculosis occur in low-income countries. Sudan is currently suffering from many factors, which may predispose for the occurrence and increase of TB infection rates, among which are the civil war in some areas and displacement of people in a search of a better life, which may enhance the occurrence and spread of many health problems, particularly the infectious diseases such as pulmonary tuberculosis. The other factors include poverty and lack of well-equipped medical centers that enable the identification and treatment of tuberculosis in its earlier stage [4]. The diagnosis of pulmonary tuberculosis generally depends on clinical history, chest X-ray, and the detection of bacteria in sputum using Ziehl-Neelsen (ZN) stain and Gene Xpert [5].

Vitamin D (Vit D) is synthesized in the skin during exposure to ultraviolet light and is also available in the diet, principally from fish [6]. Besides having a crucial role in calcium homeostasis and mineral metabolism in bone, it is known to be involved in biological functions like cell differentiation, inhibition of cell growth and immunomodulation [7]–[9]. About ten million of the world population suffers from vitamin D deficiency. Notably, several reasons can lead to vitamin D deficiency such as sunblock users, dark color skin, old age, malabsorption, obesity, some drugs, liver failure, and chronic kidney disease [10]. Vitamin D plays an important role in the host immune defense against TB infection by improving the phagocytic capacity of monocytes and macrophages [11],[12],, increasing the production of antimicrobial peptides such as cathelicidin [6], and immune modulation [13]. Vitamin D acts by binding to nuclear receptors on target cells. Therefore, both low levels of the vitamin and abnormalities in receptor structure and function may result in impairments in host immunity to the tubercle bacilli [14]. Previously, many Studies presented that the polymorphisms in the vitamin D receptor influence host susceptibility to TB [15]. Likewise, studies have shown conflicting results on the level of vitamin D in TB patients and community controls [16],[17]. Notably, serum vitamin D level varies considerably between populations and is influenced by many geographical and cultural factors [18],[19]. Although the individuals living in Sudan have a higher chance of exposure to sunlight; which is the main source of vitamin D, tuberculosis is still one of the major causes of morbidity and mortality in the country. Accordingly, this study aimed to determine the occurrence and associated factors of vitamin D deficiency in pulmonary tuberculosis patients compared to community controls at White Nile State, Sudan.

## 2. Materials and methods

### 2.1. Study design, area, and duration

This was a comparative study conducted from May 2017 to December 2018 at Kosti Teaching Hospital. This hospital locates in Kosti city of White Nile State, Sudan. It provides inpatient and outpatient services to the population of Kosti town and surrounding regions. Additionally, Kosti Teaching Hospital has a TB clinic, where pulmonary TB patients are getting their medication and further assessment during the follow-up period.

The study was approved by the Research Board of the Faculty of Medical Laboratory Sciences, University of Gezira. Further approval was obtained from the Ethics Review Committee of Kosti Teaching Hospital. The local authority and Ministry of Health in the White Nile state also provides permission. Moreover, the study steps were performed according to the declaration of Helsinki and it is ethical guidelines for the research involving human subjects, and the agreement and written informed consent were obtained from all participants.

### 2.2. Study population

The study participants consist of pulmonary tuberculosis patients and non-TB individuals regardless of gender and age. The diagnosed pulmonary tuberculosis patients based on sputum smears positive for acid-fast bacilli (AFB) along with suggestive medical history and x-ray results for pulmonary TB were included. Extra-pulmonary tuberculosis patients and those who refused to participate in this research were excluded. Accordingly, the formerly identified pulmonary tuberculosis patients and non-TB individuals as study controls (consecutive samples) were recruited. Control group were randomly selected from non-TB individuals attended to Kosti Teaching Hospital outpatient.

### 2.3. Data collection

Using a structured and pretested questionnaire, the socio-demographic data (age and sex) and tuberculosis history (for pulmonary tuberculosis group) were gathered. The questionnaire was filled by all participants from face to face interviews and following clarification of the objective of the research.

### 2.4. Sputum and blood samples collection and preparation

By standard biosafety procedures, sputum samples were collected from pulmonary TB patients in sterile sputum containers (one part of each sample was used in ZN stain and others in Gene Xpert test). Blood samples were collected from both groups and the serum was harvested immediately after centrifugation at 3000 rpm for 5 minutes and stored at −20 °C up to further processing.

### 2.5. Ziehl-Neelsen method and microscopy findings

Subsequently, a part of each sputum sample was decontaminated by using 4% sodium hydroxide and the pH of the mixture was neutralized by hydrochloric acid. Afterward, the mixture was concentrated by centrifugation at 1500–2000 rpm for 20 minutes. Later, supernatants were discarded and a double volume of distilled water was added to sediment and centrifuged again. Next, sputum smears were prepared from each specimen. Afterward, they subsequently were subjected to air dry, heat fixation, staining by Ziehl-Neelsen (ZN) method, and finally examined under 100× lens objective using light microscopy (20X Olympus, Japan) for acid-fast bacilli (AFB) as described previously [20]. Microscopy findings were stated as negative when no AFB were seen in at least 100 high power fields (HPF), exact number (1–9 AFB/100 HPF), +1 (10–99 AFB/100 HPF), +2 (1–10 AFB/HPF), or +3(more than 10 AFB/ HPF).

### 2.6. Gene Xpert analysis

Each sputum sample was re-suspended and 1ml transferred to a conical, screw-capped tube. Next, subsequently, every sputum sample was liquefied and inactivated by the addition of 2 mL of sample reagent, incubated at room temperature for 10 minutes, vortex for 10 seconds, and incubated again at room temperature for 5 minutes. Afterward, XpertMTB/RIF cartridgelid was opened and 2 ml of the liquefied sample was transferred into a sample chamber of the cartridge using a transfer pipette (The sample was dispensed slowly to minimize the risk of aerosol formation). Next, the lid was closed firmly and XpertMTB/RIF cartridge was placed into the Gene XpertDx instrument, and the instrument turned and test performed. The presence or absence of acid-fast bacilli in each sample, as well as bacillary level (Very low, Low, Medium, High) and Rifampicin susceptibility pattern for detected acid-fast bacilli strain, were reported.

### 2.7. Diagnosis of Human immunodeficiency virus infection

Human immunodeficiency virus (HIV) infection was investigated for pulmonary TB patients by a screening of patients serum for anti-HIV1/2 using rapid immunochromatographic test devices (Acon, USA) based on company guidelines. The positive test indicates by red color in test and control lines, whereas the negative by red color only in the control line.

### 2.8. Vitamin D measurement

Serum vitamin D level was measured using a 25(OH)-vitamin D3 direct Enzyme-Linked Immunosorbent Assay (ELISA) kit (MAGLUMI™, China) according to manufacturer procedure. The method utilizes a competitive ELISA technique with a selected monoclonal antibody recognizing vitamin D. It measures the serum 25(OH)-vitamin D3 concentrations in the range of 12–240 nmol/L. The interpretation of the findings was stated as described formerly [16],[21]. A 25(OH)-vitamin D3 level < 25 nmol/L defines as a severe deficiency, whereas 25–49 nmol/L as vitamin D deficiency. Likewise, the level between 50 and 75 nmol/L displays insufficiency, whereas 75–140 nmol/L represented the adequate vitamin D levels. For each sample, ELISA test was performed induplicate.

### 2.9. Statistical analysis

GraphPad Prism 5 (Graph Pad Software, La Jolla, CA, USA) was used for data analysis. The results of numerical data are expressed as mean±SEM and categorical data as number and percentage. Pearson Chi-squared test has involved in the statistical analysis of categorical data. Unpaired T-test and One-way ANOVA assessed the difference in normal distributed numerical data. For nonparametric numerical data, Mann Whitney test and Kruskal-Wallis test have used. A P-value of less than 0.05 was considered for statistical significance. ns = p ≥ 0.05, * = p < 0.05, ** = p < 0.01, *** = p < 0.001.

## 3. Results

Throughout the study duration, blood samples were collected from patients with pulmonary TB and non-TB controls, who attended to Kosti Teaching Hospital. Along with Sputum samples from pulmonary TB patients. Collectively, a total of 101 pulmonary TB patients (71 males and 30 females) and up to 100 non-TB controls (58 males and 42 females) were enrolled in this study. As seen in Table 1, the majority of TB patients were males (70.3%) and were age range of 21–40 years (38.6%). Likewise, most of them were suffered from TB relapse (36.6%); non-HIV infected individuals (99.1%) or showed a positive result for AFB (61.4%) in Gene Xpert analysis. Moreover, there is a significant difference in microscopy findings and bacillary levels of AFB, and Rifampicin (RIF) susceptibility pattern of *M. tuberculosis* strain among sputum samples of TB patients, P-values less 0.05 (Table 1).

**Figure 1.**
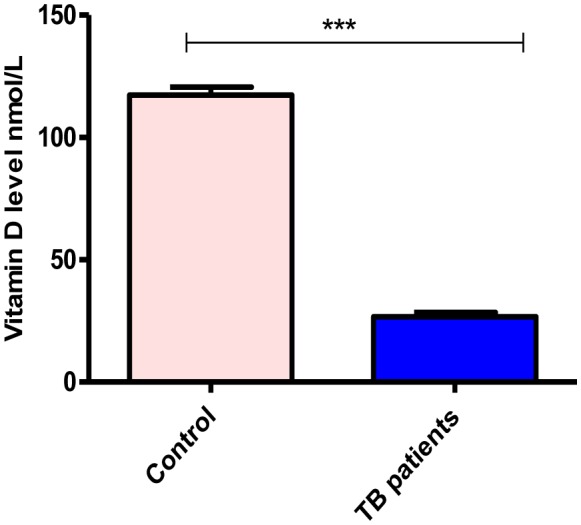
Vitamin D level among the study groups. P-value equal 0.0001.

**Figure 2.**
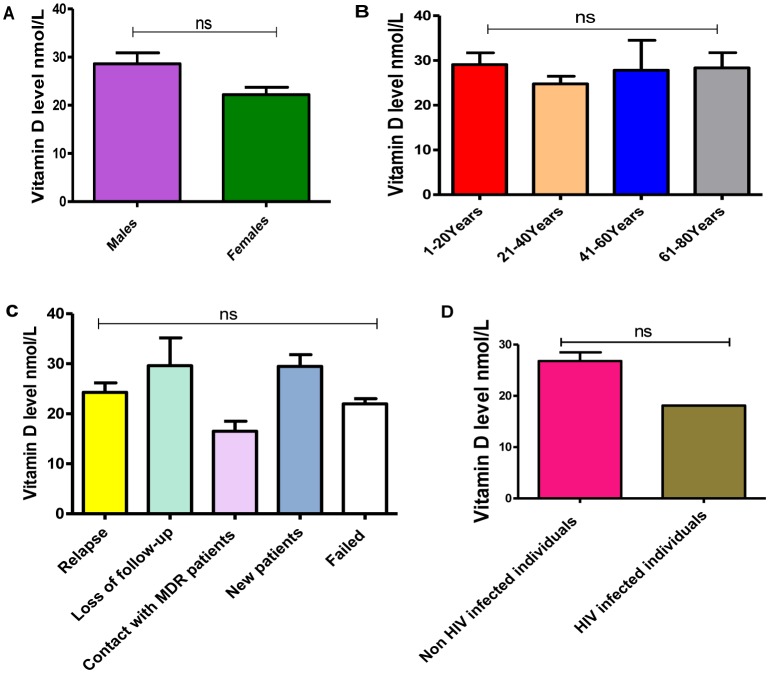
A–D: Influence of gender (A), age (B), disease history (C), and HIV infection (D) on the level of vitamin D in TB patients. ns: P-value more than 0.05.

**Figure 3.**
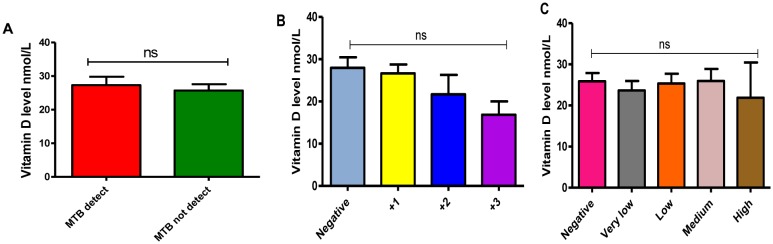
A–C: Role of Gene Xpert and microscopic findings on the evaluation of vitamin D level in TB patients. A: Positive Gene Xpert versus negative, B: Microscopy results (Positive sputum smears versus negative), C: Bacillary levels. ns: P-value more than 0.05.

**Figure 4.**
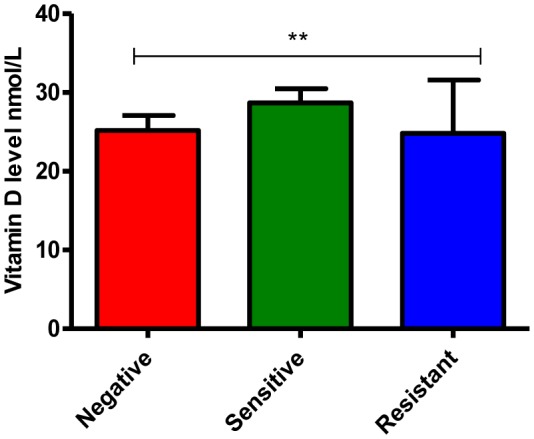
Rifampicin susceptibility patterns and vitamin D level in TB patients. P-value equal 0.0013.

In this study, we found that TB patients suffered from vitamin D deficiency. In particular, the mean of vitamin D level was significantly much lower in TB patients (26.7 ± 1.6) compared to non-TB controls (117.3 ± 3.2), P-value equal 0.0001 (Figure 1). Moreover, the average level of Vit D in TB infected individuals was non-significantly affected by gender and age (P-values more than 0.05), however, it is much lower in females 22.2 ± 1.5 (severe deficiency) compared to males 28.6 ± 2.2 (vitamin D deficiency) as well as in the individuals of age 21–40 years old 24.7 ± 1.6 (severe deficiency) compared to other age groups (Figure 2A and 2B, respectively). Likewise, Vit D level (mean) was found to be more likely to decrease up to severe deficiency level because of treatment failure (21.9 ± 1.1) and in case of infection relapse (24.2 ± 1.9) among tuberculosis patients (Figure 2C), P-values more than 0.05. On the other hand, a different average level of Vit D was also stated among those showed a positive (27.3 ± 2.4) gene Xpert result for *M. tuberculosis* compared to negative (25.7 ± 1.8) individuals (Figure 3A), P-values more than 0.05. At the microscopy level, we found a non-significantly gradual reduction of Vit D level with an increase in the number of AFB per high power field. In particular, it is dropped from vitamin D deficiency in individuals with negative sputum smear (27.9 ± 2.5) to severe deficiency (16.8 ± 3.1) in individuals with sputum smears of 3 + AFB per high power field, P-values more than 0.05 (Figure 3B). Likewise, the individuals with high bacillary levels were also showed a severe Vit D deficiency (mean = 21.9 ± 8.5) compared to patients with a negative result or have other degrees of bacillary levels, P-values more than 0.05 (Figure 3C). Moreover, there was a significant difference in the deficiency level of Vit D among Rifampicin (RIF) susceptibility pattern, P-value equal 0.0013 (Figure 4). For example, the average level of Vit D in those have infected with Rifampicin resistance strain (24.8 ± 16.7) was much lower than Rifampicin sensitive strain (28.6 ± 1.7).

**Table 1.** Characteristics of TB patients (101 individuals).

Variable		No (% from total)	Chi-squared	P-value
Gender	Male	71 (70.3)	33.287	0.000
	Female	30 (29.7)		
Age/years	1–20	20 (19.8)	13.452	0.004
	21–40	39 (38.6)		
	41–60	20 (19.8)		
	61–80	22 (21.8)		
History	Relapse	37 (36.6)	57.857	0.000
	Loss of flow-up	25 (24.8)		
	Contact with MDR	3 (3)		
	New patients	30 (29.7)		
	Failed	6(5.9)		
HIV	Positive	1 (0.9)	194.079	0.000
	Negative	100 (99.1)		
MTB	Detected	62 (61.4)	10.475	0.001
	Not detected	39 (38.6)		
Microscopy	Negative	62 (61.4)	164.158	0.000
	Exact number	0 (0)		
	+ 1	28 (27.7)		
	+ 2	7 (6.9)		
	+ 3	4 (4)		
Bacillary level	Negative	35 (34.7)	31.361	0.000
	Very low	17 (16.8)		
	Low	24 (23.7)		
	Medium	21 (20.8)		
	High	4 (4)		
Rifampicin	Negative	36 (35.6)	14.287	0.001
	Sensitive	45 (44.6)		
	Resistant	20 (19.8)		

The statistical analysis was performed using Pearson Chi-squared test. No: Number, MDR: Multi-drugs resistant, HIV: Human immunodeficiency virus, MTB: Mycobacterium tuberculosis.

## 4. Discussion

Vitamin D plays an important role in macrophage activation and restriction of mycobacterial growth. Previously, several biological studies about the effects of Vit D on the immune system of the body showed that Vit D has a definitive role in suppression of proliferation of *M. tuberculosis* [6],[11],[19]. Similarly, on triggering of toll-like receptors by molecules of the tubercle bacilli, the production of microbe-killing cathelicidin is impaired in the absence of adequate serum Vitamin D. Although the in-vivo association between Vitamin D status and tuberculosis is still a debatable issue, our study revealed that there is significant linking between vitamin D deficiency and pulmonary tuberculosis. As found in this study, the serum vitamin D level of TB patients was significantly lower than community controls. In particular, the current study finding indicated that TB patients suffer from severe vitamin D deficiency, which is consistent with studies showing vitamin D deficiency as a risk factor for developing tuberculosis [22]. Similar to the current study findings, reports from Tanzania [23], Uganda [11] and Malawi [24] found that a low BMI is associated with Vitamin D deficiency in TB patients, which may be due to the patients with low BMI usually have a little adipose tissue so they are unable to store vitamin D. Additionally, in line with our study indication, previously it has been suggested that vitamin D deficiency in TB patients might lead to impaired immune control of Mycobacteria [12],[25]. Likewise, the severe deficiency level of Vit D because of treatment failure and in case of infection relapse, gradual reduction of Vit D level with the increase in number of AFB per high power field at microscopy level, and the severe Vit D deficiency in TB patients with high bacillary level or infected with Rifampicin resistance strain in this study could also strength the relation between vitamin D deficiency and pulmonary tuberculosis.

On the other hand, however, there is no significant link between vitamin D levels and age or gender in this study, the serum level of Vit D was much lower in females than males. Likewise, TB patients age 21–40 years were found to be statistically more likely to have a lower level of vitamin D compared to others. Previously, several studies from Pakistan [26] and Ethiopia [27] showed a higher degree of vitamin D deficiency in the female gender, which might be ascribed to pregnancy and inadequate sunlight exposure. Moreover, some studies performed on an African population showed that aging was significantly associated with vitamin D deficiency [24],[27]. As well, similar findings regarding age and Vit D have also been observed in reports from Uganda [24], and in the USA [28]. This could due to fact that older people are prone to develop vitamin D deficiency because of various risk factors such as decreased dietary intake, diminished sunlight exposure, reduced skin thickness, impaired intestinal absorption, and impaired hydroxylation in the liver and kidneys [29],[30]. Although the previous studies showed similar findings, the difference in the level of vitamin D deficiency between our study and other scholars reports could be explained by the variation in study definitions of vitamin D deficiency used, techniques used for measurement of vitamin D concentrations, study area and season, study population, dietary habits, BMI of contributors, and frequencies of other co-morbidities among the respective study participants.

However, the sunlight is an important source of vitamin D [31],[32] and sun exposure is usually higher in our study area compared to Europe, the serum vitamin D level of our study subjects was found to be lower compared to subjects in Greenland [32] and West London [21]. This may be due to differences in skin pigmentation, as melanin affects the efficiency of UV radiation absorption and dark skin persons require longer time sun exposure [33]. In this regard, previously has been reported that skin pigmentation was a significant predictor variable of vitamin D deficiency [34],[35]. The difference in geographic area and population, time of the study, and food habits could also be implemented. Moreover, the variation in the prevalence and levels of vitamin D deficiency in tuberculosis patients depending on the season as reported in many studies was supported the time reason for variation [36].

## 5. Conclusions

A low vitamin D level is significantly linked to pulmonary tuberculosis. Socio-demographic characteristics of patients, history of illness, and laboratory findings are helpful in the prediction of Vit D deficiency and severity of pulmonary tuberculosis. Accordingly, our study was highlighted the TB and Vit D deficiency relationship and showed the need for further studies to a better understanding of the impact of TB on Vit D level and investigate whether vitamin D supplementation can have a role in the prevention and treatment of tuberculosis.
